# Perinatal outcomes in intrahepatic cholestasis of pregnancy with monochorionic diamniotic twin pregnancy

**DOI:** 10.1186/s12884-018-1913-z

**Published:** 2018-07-06

**Authors:** Youwen Mei, Yonghong Lin, Dan Luo, Lan Gao, Li He

**Affiliations:** Department of Obstetrics and Gynecology, Chengdu Women and Children’s Central Hospital, Chengdu City, Sichuan Province China

**Keywords:** Intrahepatic cholestasis of pregnancy, Monochorionic diamniotic twin pregnancy, Perinatal outcomes, Total bile acids

## Abstract

**Background:**

The primary aim of the study is to investigate the perinatal outcomes in intrahepatic cholestasis of pregnancy (ICP) with monochorionic diamniotic (MCDA) twin pregnancy.

**Methods:**

This study is a retrospective observational study for women with ICP and MCDA twin pregnancy. Included cases were divided into mild ICP group (10–39 mmol/L) and severe ICP group (> = 40 mmol/L), whose perinatal outcomes were compared between this two groups and whose predictors of adverse perinatal outcomes were evaluated.

**Results:**

37 cases and 21 cases are in mild and severe ICP group respectively, of which, the incidence of gestational diabetes mellitus (GDM) and iatrogenic preterm delivery in severe ICP group are higher than those in mild ICP group. Gestational age (GA) at diagnosis of ICP < 32 weeks is an independent risk factor for GA at delivery < 35 weeks and for composite adverse neonatal outcome. Total bile acids (TBA) > 40 mmol/l is an independent risk factor for meconium-stained amniotic fluid.

**Conclusion:**

For women with ICP and MCDA twin pregnancy, GA at diagnosis of ICP < 32 weeks and TBA > 40umol/L are associated with adverse perinatal outcomes.

## Background

ICP is characterized by pruritus and rising TBA level [1, 2], which could result in preterm birth, respiratory distress syndrome (RDS) and stillbirth [3–6]. Its incidence in singleton pregnancy was 0.4–15% [7], while it is two times in twin pregnancy [3, 8–10]. Monochorionic diamniotic (MCDA) twin pregnancy is one of the major type, accounting 20–25% of twin pregnancy with continuously increasing incidence [3].

In previous literature, serum TBA level has been reported to be associated with meconium stained amniotic fluid, low Apgar scores, preterm delivery and stillbirth in singleton pregnancy. However, it turns out that no literatures regarding ICP and MCDA twin pregnancy are found. The role of TBA level in MCDA twin pregnancy is not ascertained. Furthermore, the role of other variables such as transaminases and history of cholelithiasis are confusing. Therefore, our study is designed to investigate the perinatal outcomes of patients with ICP and MCDA twin pregnancy and to identify the predictors of adverse perinatal outcomes, whose conclusions may be helpful for better clinical assessments and evaluations of prognosis of patients with ICP and MCDA twin pregnancy.

## Methods

This is a retrospective observational study conducted in Chengdu’s women and children’s central hospital. Clinical data from Jan. 2013 to Jun. 2017 was extracted from electronic database after obtaining permission of accessing to clinical data and approval of Ethics Committee. Written consent for data to be used in scientific research was also acquired from patients before operation.

The including criterion consists of diagnosis of ICP and MCDA twin pregnancy according to Chinese Clinical Guideline, while the excluding criteria are as follows: the highest TBA level < 10umol/l; patients with active hepatitis, gall stone or other diseases of digestive system; patients with twin-twin transfusion syndrome, fetus growth restriction (FGR), twin reverse arterial perfusion syndrome, or twin anemia-erythrocytosis sequence and patients with severe fetal abnormalities. Were The predictors of neonatal outcomes are involved in gestational age, primipara rate, IVF-ET rate, GA at diagnosis of ICP < 32 weeks, ICP history, level of alanine aminotransferase (ALT), aspartate aminotransferase (AST), alkaline phosphatase (ALP), albumin, total bilirubin (TBIL), direct bilirubin (DBIL) and TBA. And GA at delivery, preeclampsia, GDM, oligohydramnios, placental abruption, preterm premature rupture of membrane (PPROM), chorioamnionitis, spontaneous preterm delivery, iatrogenic preterm delivery, mean birthweight, postpartum hemorrhage, meconium stained amniotic fluid and composite adverse neonatal outcome are measures for perinatal outcome.

Result of ALT, AST, ALP, albumin, TBIL and DBIL’s level were extracted from test with the highest TBA level. Diagnosis of RDS and encephalopathy were conducted by neonatologist based on Standard Clinical Guideline. Hyperbilirubinemia refers to the requirement of phototherapy, while hypoglycemia refers to the requirement of intravenous infusion. Preterm delivery means that GA at delivery is less than 37 gestational weeks. Composite adverse neonatal outcome is defined as any of the following in any neonate: neonatal intensive care unit (NICU) admission, neonatal asphyxia (Apgar score < 7 at 5 min), aspiration syndrome, neonatal respiratory distress syndrome, respiratory failure, hyperbilirubinemia, neonatal hypoglycemia, ventilator-assisted breathing, tracheal-intubation assisted breathing and encephalopathy.

For patients with ICP, the treatment protocol is similar that ursodesoxycholic acid (10–15 mg/kg qd) will be the first-line treatment. S-adenosylmethionine (0.5-1 g qd) would be prescribed for patients with elevated transaminases. Electronic fetal monitoring, blood biochemical examination, fetal ultrasound examination and Echo-Doppler detection of fetal blood flow will be performed regularly. The indications for iatrogenic preterm delivery are fetal distress, poor treatment effect or GA > 35 weeks.

IBM SPSS Statistics for Windows, Version 19.0 is used for data analysis. Student’s T test or Mann-Whitney U test are used to assess continuous variables according to its distribution. χ2 analysis is utilized to assess categorical variables. Logistic regression analysis was performed to identify predictors of adverse perinatal outcomes. *P* < 0.05 indicates significant differences.

## Results

There are 58,862 cases of pregnant women delivering babies in our hospital from Jan. 2013 to Jun. 2017, among which there were 1601 cases of twin pregnancy, 350 cases of ICP with twin pregnancy, and 64 cases of ICP with MCDA pregnancies. Three cases were excluded because of twin-twin transfusion syndrome, and other three cases were because of FGR. Finally, 37 cases and 21 cases were included respectively in mild ICP group and severe ICP in the final analysis, in all which, people delivered babies by caesarean section.

Continuous variables do not normally distribute except mean birth weight. There is no significant difference in baseline characteristics in terms of GA, primipara rate, IVF-ET rate and level of ALT, AST, ALP, albumin, TBIL, and DBIL between two groups (Table 1). The incidence of GDM and iatrogenic preterm delivery in severe ICP group are significantly higher than those in mild ICP group (*P* < 0.05). Although the rate of meconium-stained amniotic fluid is higher in severe ICP group than those in mild ICP group, the difference is not statistically significant. Other outcome measures are not dissimilar in both groups (Table 2). One case shows severe ICP and GDM suffered single fetal death in our study. In this case, one fetal was found dead at 31 + 4 gestational weeks, but cesarean section was performed at 31 + 5 weeks to be found with one alive neonate. Only 18 patients had complete course of blood test, among which, 17 patients’ TBA level had decreased after treatment, 6 patients had meconium stained amniotic fluid, and one patient had suffered severe asphyxia. However, there was no meconium stained amniotic fluid and asphyxia in the only one patient whose TBA level kept on increasing.

**Table 1:** Baseline characteristic of both groups

	Mild ICP group	Severe ICP group	P
Mean maternal age(y,±SD)	27.65 ± 4.61	27.29 ± 3.62	0.819
BMI	22.8 ± 2.61	22.74 ± 3.38	0.744
Nulliparous	28(75.7%)	14(66.7%)	0.546
IVF-ET	5(13.5%)	1(4.8%)	0.402
GA at diagnosis(weeks,±SD)	32.92 ± 2.51	31.8 ± 5.48	0.878
GA at diagnosis< 32 weeks	12(32.4%)	8(38.1%)	0.776
ALT(U/L,±SD)	173.8 ± 162.1	217.9 ± 156.6	0.314
AST(U/L,±SD)	136.2 ± 128.8	144.7 ± 94.5	0.361
ALP(U/L,±SD)	352.3 ± 105.7	322 ± 125.1	0.304
Albumin(g/L,±SD)	31.1 ± 4.1	31.9 ± 3.7	0.476
TBIL(umol/l,±SD)	14.5 ± 7.3	19.4 ± 15.2	0.313
DBIL(umol/l,±SD)	8.1 ± 5.3	12.8 ± 13.8	0.418
TBA(umol/L,±SD)	20.4 ± 6.3	75.3 ± 33.8	0
ICP history	1(2.7%)	0	1

**Table 2:** Maternal and neonatal outcomes of both groups

	Mild ICP group(n,%)	Severe ICP group(n,%)	P	OR
GDM	5(13.5%)	8(38.1%)	0.049	3.938(1.084–14.307)
Pre-eclampsia	2(5.4%)	2(9.5%)	0.615	1.842(0.24–14.138)
Hypothyroidism	2(5.4%)	1(4.8%)	1	0.875(0.075–10.268)
placental abruption	1(2.7%)	0	1	0.632(0.518–0.77)
PPROM	2(5.4%)	0	0.53	0.625(0.51–0.766)
postpartum hemorrhage	2(5.4%)	0	0.53	0.625(0.51–0.766)
meconium stained amniotic fluid	18.9%	42.9%	0.069	3.214(0.975–10.6)
GA at delivery (weeks,± SD)	34.95,1.5	34.7,1.4	0.408	
GA at delivery< 35 weeks	29.7%	42.9%	0.392	1.773(0.581–5.408)
spontaneous preterm delivery	13(35.1%)	4(19.1%)	0.245	0.434(0.121–1.564)
iatrogenic preterm delivery	20(54.1%)	17(81%)	0.05	3.613(1.018–12.82)
iatrogenic preterm delivery with GA < 35 weeks	4(10.8%)	6(28.6%)	0.089	3.3(0.81–13.445)
Mean birth weight(g,± SD)	2223.4316.8	2177.1396.6	0.628	
NICU	25(67.6%)	13(61.9%)	0.776	0.78(0.255–2.385)
neonatal asphyxia	5(13.5%)	3(14.3%)	1	1.067(0.228–4.993)
aspiration syndrome	2(5.4%)	1(4.8%)	1	0.875(0.075–10.268)
neonatal respiratory distress syndrome	2(5.4%)	2(9.5%)	0.615	1.842(0.24–14.138)
pneumonia	9(24.3%)	9(42.9%)	0.237	2.333(0.743–7.332)
respiratory failure	4(10.8%)	2(9.5%)	1	0.868(0.145–5.195)
hyperbilirubinemia	2(5.4%)	1(4.8%)	1	0.875(0.075–10.268)
hypoglycemia	3(8.1%)	0	0.547	0.618(0.502–0.761)
ventilator-assisted breathing	3(8.1%)	3(14.3%)	0.657	1.889(0.345–10.332)
tracheal intubation assisted breathing	2(5.4%)	2(9.5%)	0.615	1.842(0.24–14.138)
encephalopathy	3(8.1%)	0	0.547	0.618(0.502–0.761)
still-birth	2(5.4%)	1(4.8%)	1	0.87(0.075–10.268)
adverse composite neonatal outcome	8(67.6%)	13(61.9%)	0.438	0.78(0.255–2.385)

The risk factors of major perinatal outcomes are as follows. DBIL level > 7 mmol/l, GDM and GA at diagnosis of ICP < 32 weeks are related with GA at delivery < 35 weeks in χ2 analysis, while GA at diagnosis of ICP is an independent risk factor in logistic regression analysis. TBA level > 40umol/l is related with meconium stained amniotic fluid in both χ2 analysis and logistic regression analysis. DBIL level > 7 umol/l, GA at diagnosis< 32 weeks and ALP level > 400 U/L are associated with composite adverse neonatal outcomes in χ2 analysis, while GA at diagnosis of ICP < 32 weeks is an independent risk factor in logistic regression analysis (Tables 3, 4 and 5).

**Table 3:** Predictors of GA at delivery< 35 weeks

GA at delivery< 35 weeks	chi square value	P	OR	95%CI lower	95%CI upper
DBIL> 7 umol/l	7.917	0.01	5.385	1.587	18.264
GDM	5.429	0.043	4.4	1.201	16.114
GA at diagnosis< 32 weeks	22.182	0.000	19.800	4.973	78.837
binary logistic analysis	B	P	adjusted OR^a^	95%CI lower	95%CI upper
GA at diagnosis< 32 weeks	2.917	0	18.48	4.611	74.068

“a”means adjusted for age, nulliparous, IVF-ET,GA at diagnosis< 32 weeks, ALT> 200 U/L,AST > 200 U/L, ALP > 400 U/L, TBIL> 17.1umol/l, DBIL> 7umol/l, TBA > 40 umol/l

**Table 4:** Predictors of meconium-stained amniotic fluid

	chi square value	P	OR	95%CI lower	95%CI upper
TBA > 40 umol/l	3.843	0.069	3.214	0.975	10.6
binary logistic analysis	B	P	adjusted OR^b^	95%CI lower	95%CI upper
TBA > 40 umol/l	1.322	0.035	3.75	1.095	12.842

“b”means adjusted for age, nulliparous, IVF-ET,GA at diagnosis< 32 weeks,ALT> 200 U/L,AST > 200 U/L, ALP > 400 U/L, TBIL> 17.1umol/l, DBIL> 7umol/l

**Table 5:** Predictors of adverse composite neonatal outcome

	chi square value	P	OR	95%CI lower	95%CI upper
DBIL> 7 umol/l	3.728	0.094	3.063	0.963	9.736
GA at diagnosis< 32 weeks	5.129	0.04	4.587	1.15	18.306
ALP > 400 U/L	4.634	0.061	0.276	0.083	0.920
binary logistic analysis	B	P	adjusted OR^c^	95%CI lower	95%CI upper
GA at diagnosis< 32 weeks	1.463	0.039	4.317	1.076	17.318

“c” means adjusted for means adjusted for age, nulliparous, IVF-ET, GA at diagnosis< 32 weeks, ALT> 200, AST > 200,ALP > 400, TBIL> 17.1,DBIL> 7, TBA > 40 umol/l

## Discussion

Maximum TBA level was used to determine the severity of ICP in previous literature [3, 11–13]. Due to various impact of ICP on perinatal outcomes, composite adverse neonatal outcome was created and defined to evaluate the perinatal outcomes of ICP [14, 15]. And our study drew on these methodologies.

A large number of studies about single pregnancy had found that the higher TBA level exists, the higher rate of adverse perinatal outcomes [3, 11–13, 16]. The study of LIU Xiaohua [17] and SHAN Dan [18] about twin pregnancy also made similar conclusions. However, our study reveals that the major perinatal outcomes is similar between severe ICP group and mild ICP group, which is consistent with the studies of Gonzalez MC [10] and Andrea Y. Lausman [19]. The reason is probably that the rigorous monitoring, standardized treatment, and timely termination of pregnancy have reduced the harm of high TBA level on neonates. The rate of iatrogenic preterm birth in severe ICP group is higher than that in mild ICP group presenting in our study, which is consistent with the studies of SHAN Dan [18] and Maria C. Estiu [12]. The explanations are as follows: 1, Higher TBA level results in frequent abnormal fetal heart racing or Echo-Doppler detection of fetal blood flow, which increases the rate of fetal distress detection. 2. Higher TBA level is considered to be associated with higher rate of adverse perinatal outcomes by obstetrician who would be liable to terminate pregnancy earlier.

SHAN Dan’ study concluded the incidence of GDM increased in twin pregnancy with ICP [18], while our study revealed the incidence of GDM in severe ICP group is higher than that in mild ICP group. The mechanism may be that bile acids could increase the expression of farnesoid receptors (FXR), and destroy homeostatic pathways for glucose balance system [20]. It was also found the rate of preeclampsia increased in ICP patients with twin pregnancy in previous studies [21]. However, the preeclampsia rate was not different between mild ICP group and severe ICP group in our study. But the sample size of preeclampsia in our study is too small (four cases) to make a conclusion.

According to SHAN Dan [18] and R. Madazli’s [22] studies, the rate of adverse perinatal outcomes would increase in patients with earlier ICP onset gestational age, which is also the same result found in our study. Therefore, more concerns should be given to these patients. Our study’s result is in keeping with previous literature, which makes the conclusion that higher TBA level leads to higher rate of meconium stained amniotic fluid [14]. The reason may be that elevated bile acids altered colonic motility [7]. Stillbirth with ICP and twin pregnancy usually occurs at 33–35 weeks [17], and stillbirth’s rate increases with higher TBA levels or combination with GDM or other complications [13, 23]. In our study, there was only one case shows dead fetal, in which severe ICP and GDM are suffered.

It was recommended that 36 weeks was the proper gestational age to terminate pregnancy in ICP patients with singleton pregnancy [24, 25]. However, there was no recommendation for twin pregnancy with ICP. In our study, the mean GA at delivery was 34.7 + − 1.4 weeks in severe ICP group, while 34.95 + − 1.5 weeks in mild ICP group, which may provide some suggestions about timing of delivery for these patients.

Advantages of our study are as follows. 1.Our study is one of the largest studies assessing MCDA twin pregnancy with ICP. 2. It only includes patients of MCDA twin pregnancy, avoiding bias of different chorionicity. 3. A wide variety of factors are included, leading to comprehensive conclusions. The major limitation of our study is that it is a retrospective and observational study. Some patients’ conditions were not fully documented. As a result, we could not evaluate outcomes based on change of blood test’s result. Furthermore, the concept of adverse composite neonatal outcome was drawn from previous studies but not the same. Therefore, comparison with other studies should be interpreted with caution.

## Conclusion

GA at diagnosis of ICP < 32 weeks and TBA > 40umol/L are associated with adverse perinatal outcomes in our study. Further large prospective trials are required to identify the predictors of adverse neonatal outcomes and the optimal timing of delivery for patients with ICP and MCDA twin pregnancy.

## Abbreviations

ALP: Alkaline phosphatase
ALT: Alanine aminotransferase
AST: Aspartate aminotransferase
DBIL: Direct bilirubin
DCDA: Dichorionic diamniotic
FXR: Farnesoid receptors
GA: Gestational age
GDM: Gestational diabetes mellitus
ICP: Intrahepatic cholestasis of pregnancy
MCDA: Monochorionic diamniotic
MCMA: Monochorionic monoamniotic
NICU: Neonatal intensive care unit
PPROM: Preterm premature rupture of membrane
TBA: Total bile acid
TBIL: Total bilirubin
